# Religious Moderation Dataset of Senior High School Students in Indonesia

**DOI:** 10.5334/jopd.163

**Published:** 2026-10-12

**Authors:** Dinar Pratama, Imam Alfikri Pratama, Janawi Janawi, Syatria Adymas Pranajaya, Sumar Sumar, Syarifah Syarifah, Fazrul Sandi Purnomo

**Affiliations:** 1 Institut Agama Islam Negeri Syaikh Abdurrahman Siddik Bangka Belitung, Indonesia; 2 UIN Sultan Aji Muhammad Idris Samarinda, Indonesia; 3 UIN Syarif Hidayatullah Jakarta, Indonesia

**Keywords:** Religious Moderation, National Commitment, Tolerance, Anti-Violence, Accommodation of local culture

## Abstract

This article presents a comprehensive dataset on attitudes towards religious moderation among 4.197 secondary school students in the Bangka Belitung Islands Province, Indonesia. Data collection was conducted online via Google Forms between November and December 2022. The participants represent a homogeneous developmental group in late adolescence, with an age range generally between 15 and 19 years and a mean age of approximately 16.8 years. The gender distribution of the sample comprised 1.627 males and 2.570 females. This dataset measures four key dimensions of religious moderation (national commitment, tolerance, anti-violence, and accommodation of local culture). This paper presents a large-scale dataset on adolescent attitudes toward religious moderation, validated using the Rasch model to ensure high psychometric quality and reliability. This dataset represents a valuable resource for researchers in the fields of educational psychology, the sociology of religion, and policymakers. It can be used to identify potential risks of extremism, design evidence-based character education curricula, or serve as a baseline for longitudinal studies on religious harmony among the younger generation in multicultural societies.

## (1) Background

The Institute for Economic & Peace (IEP) report (Institute for Economic & Peace, 2025) still shows the high issue of radicalism-terrorism in the name of religion, both at the global and national levels. Indonesia, nationally, is in the medium category on the Global Terrorism Index (GTI) (4.17) in 2025 (Institute for Economic & Peace, 2025). One group that is vulnerable to radical terrorism is students, especially high school students (Subchi et al., 2022). An immoderate understanding of religious practice can threaten harmony among religious believers (Latifa et al., 2022). The context of this dataset collection is based on the vulnerability of high school students to exposure to radical terrorism (Subchi et al., 2022), which is also influenced by the diversity and sensitivity of religious life in Indonesia (Mazya et al., 2024). This vulnerability is even higher when there is no effort from various parties to understand how students’ religious practice behavior (Mangestuti & Aziz, 2023). Moreover, most high schools in Indonesia have religious extracurricular activities that are sometimes not optimally monitored. Students’ religious understanding may start from their involvement in these studies. The results of previous studies related to students’ religious activities in schools highlight the importance of monitoring the material presented in these religious studies. Departing from this fact, one of the preventive efforts that can be made is to measure students’ religious understanding and attitudes. The data from these measurements then becomes important information for interested parties to explore students’ religious attitudes.

### 1.1 Theoretical background of religious moderation

In the context of religious practice, the term moderate can be understood as a democratic attitude, supporting human rights, upholding gender equality, and promoting worship. A moderate attitude in religion is also an attitude of respect for the beliefs and practices of other religions. (Zakiyah et al., 2023). Religious moderation refers to a balanced attitude in practicing religious teachings and respecting other religions. In addition, the concept of religious moderation has one main goal, which is to create harmony among religious communities (Ahmad, 2021). In Indonesia, this moderate practice of religion is known as religious moderation. Because Indonesia is a country that recognizes religious, ethnic, racial, and cultural diversity, the Ministry of Religious Affairs of the Republic of Indonesia felt it necessary to intervene by creating the concept of religious moderation. There are at least four dimensions of religious moderation developed by the Ministry of Religious Affairs of the Republic of Indonesia, namely 1) commitment to nationality, 2) tolerance, 3) anti-violence, and 4) accommodation of local cultures (Ministry of Religious Affairs, 2019).

The theoretical framework for the four dimensions used in this study is based on the conceptualisation developed by the Ministry of Religious Affairs of the Republic of Indonesia (2019). Moderatism is defined not as a compromise on religious dogma, but as a balanced perspective and practice of religion (*wasathiyyah*). These four dimensions were chosen as key indicators because they represent the meeting point between religious values and civic obligations in a pluralistic society.

**National commitment**, loyalty to the foundations of the state, the constitution, and regulations, as well as a willingness to implement religious teachings within the framework of national consensus and Indonesian social life (Fitri et al., 2025; Mazya et al., 2024; Subchi et al., 2022). Psychologically, this dimension provides a superordinate social identity, “we as Indonesians,” which encompasses various religious and ethnic identities. This superordinate identity reduces the potential for intergroup conflict, as religious differences are no longer understood as a threat, but as variations within the same political community (Brown, 2017). Research on religiosity and nationalism among Indonesian Muslims shows that strong religiosity can go hand in hand with, and even strengthen, nationalism, rather than the opposite (Subchi et al., 2022; Suprapto et al., 2025).

**Tolerance**, readiness to respect differences in beliefs, allow space for others to express their religion, refrain from discrimination, and accept the peaceful co-existence of different views (Adnan et al., 2024; Fauziyah & Hidayati, 2023; Pajarianto et al., 2022). In the context of psychology, this relates to a reduction in prejudice, an increase in empathy, and openness towards “the Others” (Anas et al., 2025; Widyawati et al., 2025). Tolerance is at the affective-cognitive core of religious moderation. Without it, national commitment easily falls into majority exclusivism, and cultural accommodation can be seen as mere formality without inner acceptance (Pajarianto et al., 2022). Studies in Indonesia related to tolerant major cities such as Salatiga show that public policy, education, and structured social interaction can strengthen citizens’ ability to accept other groups positively (Anas et al., 2025).

**Anti-violence**, rejection of ideologies and radical actions that seek to change the socio-political system through verbal, physical, or psychological violence in the name of religion; prioritising dialogue, deliberation, and peaceful resolution (Fauziyah & Hidayati, 2023; Oktaviana & Albina, 2025). This dimension relates to aggression control, rejection of moral justification for violence, and preference for peaceful conflict resolution strategies (dialogue, deliberation, mediation) (Widyawati et al., 2025). Within the framework of religious moderation promoted by the Ministry of Religious Affairs, anti-violence is reinforced by the principles of justice and balance (al-‘adl, al-tawazun), which reject both extremism (ghuluw) and neglect of obligations (tafrith) (Nurrohim et al., 2024). Tolerance and commitment to national unity underpin this anti-violence dimension: the stronger the appreciation of diversity and loyalty to national unity, the less likely a person is to view violence as a legitimate means of pursuing religious aspirations (Ropi, 2019).

**Accommodative of Local Culture** describes openness to integrating local traditions and wisdom into religious practices as long as they do not conflict with core teachings (Fitri et al., 2025; Fuadi et al., 2024). This dimension requires cognitive flexibility and the ability to manage dual identities, namely as a religious adherent and as a member of a particular cultural community (Fuadi et al., 2024). At the practical level, research on local wisdom, such as kinship and togetherness values, shows that local culture can be an effective medium for transmitting values of moderation and interfaith tolerance within families and communities (Pajarianto et al., 2022).

Theoretically, these four dimensions are interrelated, forming a construct of religious moderation. Recent empirical evidence suggests that these dimensions, such as tolerance, can also serve as critical predictors of peaceful coexistence and reduced religious violence (Basedau et al., 2021). National commitment provides a framework for shared identity and goals, tolerance regulates how other groups are viewed and treated, anti-violence functions as a behavioural barrier, and accommodation of local culture bridges the tension between religious norms and socio-cultural realities. Understanding these dimensions is crucial because threats to identity and perceptions of superiority are often key factors that trigger religious conflict (Basedau et al., 2021). In the context of psychology, this construct can be positioned as a multidimensional religious attitude that mediates the influence of religiosity on social outcomes, such as acceptance of diversity, rejection of radicalism, and constructive involvement in society. Research shows that dimensions of religiosity (intellectual, ideological, experiential, public, and private practices) contribute positively to religious moderation scores: the more central religion is to the self, the greater the potential for moderation when accompanied by inclusive understanding. Thus, religious moderation is not a weakening of religion, but rather a strengthening of the quality of religiosity so that it is in harmony with the demands of peaceful living in a pluralistic society.

## (2) Methods

### 2.1 Study design

The data presented in this article are cross-sectional and focus on students’ attitudes toward religious moderation. The participants in this study were secondary school students in the Bangka Belitung Islands Province, Indonesia. The students completed an online survey administered via Google Forms. The main focus of this survey was to obtain data covering four dimensions of religious moderation, namely 1) national commitment, 2) tolerance, 3) anti-violence, and 4) accommodation of local culture.

### 2.2 Time of data collection

Data collection was conducted from November to December 2022. The survey was completed in stages at all schools included in the sample.

### 2.3 Location of data collection

Survey data were collected from seven districts/cities in the province of Bangka Belitung, Indonesia, namely Bangka, West Bangka, South Bangka, Central Bangka, Belitung, East Belitung, and Pangkalpinang.

### 2.4 Sampling, sample, and data collection

Stratified random sampling techniques were used to collect data. The target sample for this study was 4.234 students from various schools. The stratified random sampling was conducted based on two main criteria, geographical distribution (covering 7 regencies or cities in Bangka Belitung) and school type. To increase the response rate, small incentives in the form of souvenirs were provided to a randomly selected subset of respondents. Souvenirs were provided as appreciation tokens for participation. These souvenirs were distributed randomly to a small subset of respondents after the completion of the survey to avoid influencing response behavior. A total of 100 recipients were selected using a random lottery system from the pool of completed responses. From this target sample, a total of 4.197 valid questionnaires were returned and included in the final dataset, representing a high overall response rate of 99.1%. To facilitate data collection and follow-up, the research team sent reminders by communicating directly with school administrators and teachers responsible for coordinating the data collection process at each target school. The demographic characteristics of the participants are presented in Table 1.

**Table 1:** Identity and Demographic Variables (n = 4.197).


VARIABLE	CATEGORY	FREQUENCY	PERCENTAGE (%)

Gender	Male	1.627	38.8%

	Female	2.570	61.2%

Religion	Islam	3.512	83.7%

	Buddha	316	7.5%

	Protestant	157	3.7%

	Catholic	128	3.0%

	Confucianism	82	2.0%

	Hindu	2	0.0%

Parents’ Employment Status	Working	2.627	62.6%

	Not Working	1.570	37.4%

Type of School	High School	1.921	45.8%

	Islamic High School	1.551	37.0%

	Vocational High School	725	17.3%

School Status	Public	2.994	71.3%

	Private	1.203	28.7%

City of Origin	Bangka	780	18.6%

	West Bangka	825	19.7%

	South Bangka	572	13.6%

	Central Bangka	368	8.8%

	Belitung	660	15.7%

	East Belitung	431	10.3%

	Pangkalpinang	561	13.4%


The sample size for this study was not determined based on power analysis (such as 1-β), which is commonly used to test inferential hypotheses. Instead, because the main objective of this study was to describe the level of religious moderation among students, the sample size was determined based on the desired estimation precision. This survey used the Cocran (Tengeh & Talom, 2020) sample estimation formula for large populations, with a confidence level of 95% (equivalent to Z = 1.96) and a margin of error (e) of 5% (0.05), assuming the most conservative population variance (p = 0.5). The minimum sample size required is 385. Based on these calculations, the minimum sample size required to achieve ± 5% precision is 385 participants. Our final sample size of 4197 participants exceeded this minimum threshold, ensuring that our findings are highly precise and can be generalized with 95% confidence.

Although the minimum sample size required for estimation accuracy was 385 respondents, the research team collected responses from all available students in participating schools. As the survey was conducted at the class level in each participating school, some classes were invited to participate after institutional permission was granted. This approach resulted in a larger dataset (N = 4197), thereby increasing the sample’s representativeness and enhancing the potential for secondary analysis by other researchers (Langener et al., 2024).

Scale filling by respondents was done online through a Google Form. The selection of Google Forms as a medium for collecting response data is because this survey uses a large sample (4197), so that the data collection process will be more effective and efficient. The responses were exported to Microsoft Excel for coding. After the raw data is obtained, the next process is data processing and analysis. At this stage, the data is processed and analyzed using JASP (version 0.95.1.0) and WINSTEPS (version 3.69.1).

To minimize duplicate participation, the online survey settings limited responses to one submission per user account. Additionally, responses were screened during the data-cleaning process to identify potential duplicate entries based on time stamps and identical response patterns.

In addition, the survey also collected responses from students regarding religious moderation attitudes, consisting of 1) national commitment, 2) tolerance, 3) anti-violence, and 4) accommodation of local culture. In addition, in this article, the statement items contained in the student religious moderation scale are used to measure respondents’ perceptions related to religious moderation attitudes. Using a Likert scale (5 options) from strongly disagree to agree strongly.

### 2.5 Materials/Survey instruments

#### Instrument Structure and Data Description

Data were collected using 36 statement items on a religious moderation scale consisting of favorable and unfavorable items. Using a Likert scale (1–5 scale) from strongly disagree to strongly agree. The scale structure consists of 4 dimensions, namely,

- National Commitment, an attitude of accepting Pancasila as the state ideology.
- Tolerance is an attitude of accepting, appreciating, and respecting differences.
- Anti-violence is an attitude of rejecting radical actions in the name of religion.
- Accommodating local culture, can accept local and national cultural practices that do not conflict with religious teachings.

The measurement instrument comprised 36 items across four dimensions of religious moderation. The full list of item statements, along with their dimensional classification and directional coding, is provided in Table 2.

**Table 2:** Item Statements for the Religious Moderation Scale.


NO	DIMENSION	ITEM STATEMENT	DIRECTION

A	National Commitment	1. Pancasila is final as the state ideology and is appropriate for Indonesia.	Favorable

		2. The various problems occurring in Indonesia today are because Pancasila is not a suitable ideology.	Unfavorable

		3. Amidst the nation’s problems, the belief I hold is more appropriate to be the state ideology.	Unfavorable

		4. The religion I believe in is in harmony with the Pancasila ideology.	Favorable

		5. The values of Pancasila need to be practiced in daily life to build national commitment.	Favorable

		6. As Islam is the majority religion in Indonesia, an Islamic State should be established as the state ideology.	Unfavorable

		7. I am not very proud to be an Indonesian citizen because we lag behind in many aspects.	Unfavorable

		8. Regardless of the nation’s condition, I always strive to contribute according to my abilities.	Favorable

		9. I prioritize deliberation (musyawarah) in solving problems.	Favorable

		10. For me, it is more important to fight for personal and family interests than for the interests of the group or the state.	Unfavorable

B	Tolerance	11. I accept the existence of differences in ethnicity, race, religion, and between groups.	Favorable

		12. I will not support a regional/national leader who is not from my religion.	Unfavorable

		13. Every citizen has the same right to practice the religion they believe in.	Favorable

		14. In social interactions, I only choose friends who share my faith.	Unfavorable

		15. I believe my religion’s teachings are the most correct, but I still respect other religions.	Favorable

		16. The existence of various religions in Indonesia has the potential to cause national division.	Unfavorable

		17. I am willing to cooperate with anyone, regardless of their religious background.	Favorable

		18. Religious differences are not a barrier to helping one another for the common good.	Favorable

		19. I feel uncomfortable when people of other faiths build their places of worship in my neighborhood.	Unfavorable

		20. I will not prohibit adherents of other religions from performing their worship.	Favorable

C	Anti-Violence	21. I agree with coercive actions against groups considered heretical.	Unfavorable

		22. I am willing to engage in conflict with those who have different beliefs.	Unfavorable

		23. I choose dialogue to resolve differences in religious understanding.	Favorable

		24. Violence in the name of religion cannot be justified under any circumstances.	Favorable

		25. When I see injustice, I think it is better to take matters into my own hands rather than leave it to law enforcement.	Unfavorable

		26. In my opinion, the act of damaging places of worship can be justified.	Unfavorable

		27. I prefer to avoid any form of violence that could harm others.	Favorable

		28. I believe that every problem can be solved without violence.	Favorable

		29. Disbanding the religious activities of others is permissible if they are not in accordance with my religion.	Unfavorable

		30. I try to persuade friends to commit acts of violence against a group of people who insult my beliefs.	Unfavorable

D	Accommodative of Local Culture	31. The teachings of my religion provide space to accept good cultures or traditions that have long existed in society.	Favorable

		32. All local cultures or traditions that develop in society are contrary to the teachings of my religion.	Unfavorable

		33. I always try to appreciate and respect every culture or tradition that develops in Indonesia.	Favorable

		34. In my opinion, all local traditions that develop in society are not in accordance with the teachings of my religion.	Unfavorable

		35. I believe that the local cultures or traditions that develop in society are one of the forces that strengthen national unity.	Favorable

		36. Disasters or calamities that occur in this nation are a reprimand from God because people mix religious teachings with local culture or traditions.	Unfavorable


These items were developed based on the conceptual framework of the Ministry of Religious Affairs (2019) and have undergone content validation by three experts in the fields of psychometrics and sociology. For the purposes of this data paper, the original items in Indonesian were translated into English using a back-translation procedure carried out by professional translators to ensure semantic equivalence.

### 2.6 Quality Control

A pilot study was conducted with 122 respondents in three schools to evaluate the time needed to complete the survey (between 15 and 20 minutes on average). This pilot study also aimed to assess the clarity of items on the religious moderation scale and their suitability for respondents in various provinces. The results of our pilot study were satisfactory, and no further revisions to the scale items were necessary.

To ensure valid and reliable measurement results, the data were analyzed using Rasch modeling through WINSTEPS Version 3.69.1 software. The results of this analysis produced several important parameters such as unidimensionality (Slocum-Gori & Zumbo, 2011), item and person reliability (Ehrenthal et al., 2023), and item fit (Zhang et al., 2019).

In evaluating the psychometric properties of the scale, Rasch modelling was applied using WINSTEPS Version 3.69.1. Rasch analysis was employed because it allows the transformation of ordinal Likert-scale responses into interval-level measurements while simultaneously estimating item difficulty and respondent ability on a common logit scale. (Al-Jaishi et al., 2026). This approach is widely used in scale validation studies as it provides detailed diagnostics for item functioning and measurement quality.

In this study, both Infit Mean Square (MNSQ) and Outfit Mean Square (MNSQ) statistics were examined to evaluate item fit. Infit statistics are sensitive to unexpected responses to items that are targeted near a respondent’s ability level, whereas Outfit statistics are more sensitive to unexpected responses on items that are far from a respondent’s ability level (Qin et al., 2024; Welter et al., 2024). Because the present study involves a large and heterogeneous sample of students, particular attention was given to Outfit MNSQ values, as these statistics are effective in identifying irregular response patterns that may occur when respondents answer items that are either too easy or too difficult relative to their level of religious moderation. Following common guidelines in Rasch measurement, item fit was evaluated using the recommended range of 0.5 to 1.5 for Mean Squared statistics, indicating productive measurement for rating scale data. Items falling outside this range were further examined for potential misfit and considered for removal from the scale. The summary of the psychometric analysis results is presented in Table 3.

**Table 3:** Summary of Psychometric Analysis Results.


PSYCHOMETRIC PARAMETER	STATISTIC	ANALYSIS RESULT	THRESHOLD

Unidimensionality	Raw Variance Explained by Measures	42.4%	>40% (Excellent)

	Unexplained Variance in 1st Contrast	2.2%	<15% (Good)

Reliability	Person Reliability	0.86	>0.80 (Excellent)

	Item Reliability	1.00	>0.90 (Excellent)

	Cronbach Alpha (KR-20)	0.87	>0.80 (Excellent)

Item Fit	Outfit MNSQ	B15 (1.59), C21 (1.59), C23 (1.89)	0.5 – 1.5

	Outfit ZSTD	B15 (9.9), C21 (9.9), C23 (9.9)	–2.0 – +2.0

	Point-Measure Correlation	B15 (0.26), C21 (0.22) C23 (0.26)	>0.3


Based on the results of the initial evaluation of Rasch analysis on item fit parameters (Table 2), 3 items (B15, C21, C23) were detected as items that did not fit the model because they did not enter the threshold value of outfit MNSQ, outfit ZSTD, and Point-Measure Correlation as Rasch parameters. The results of the evaluation of item fit in the initial analysis were used as a basis for conducting a second analysis by eliminating these 3 items. A comparison between the initial and final psychometric analysis results is presented in Table 4.

**Table 4:** Comparison Table of Psychometric Analysis Results with Thresholds.


PSYCHOMETRIC PARAMETER	THRESHOLD/CRITERIA	INITIAL ANALYSIS (36 ITEMS)	FINAL ANALYSIS (33 ITEMS)	NOTES

**Reliability**				

Person Reliability	>0.80 (Excellent)	0.86	0.87	Improved

Item Reliability	>0.90 (Excellent)	1.00	1.00	Remained Excellent

Cronbach Alpha (KR-20)	>0.80 (Excellent)	0.87	0.88	Improved

**Unidimensionality**				

Raw Variance Explained	>40% (Excellent)	42.4%	44.3%	Construct strength improved


The results of item evaluation in the second stage of 33 statement items obtained an increase in the parameters of person reliability, Cronbach’s alpha (KR-20), and Unidimensionality.

While visually, it can be seen that the distribution of items and respondents’ answers, as in Figure 1, shows the distribution of items ranging from those that are easy for respondents to agree with to the most difficult items for respondents to agree with. The Wright Map reveals that most items are clustered around the mean of the person distribution. This indicates that the instrument provides high measurement precision for the majority of the student population who hold moderate views, although it suggests a need for additional extreme items in future developments to better differentiate respondents at the very high or very low ends of the spectrum.

**Figure 1:**
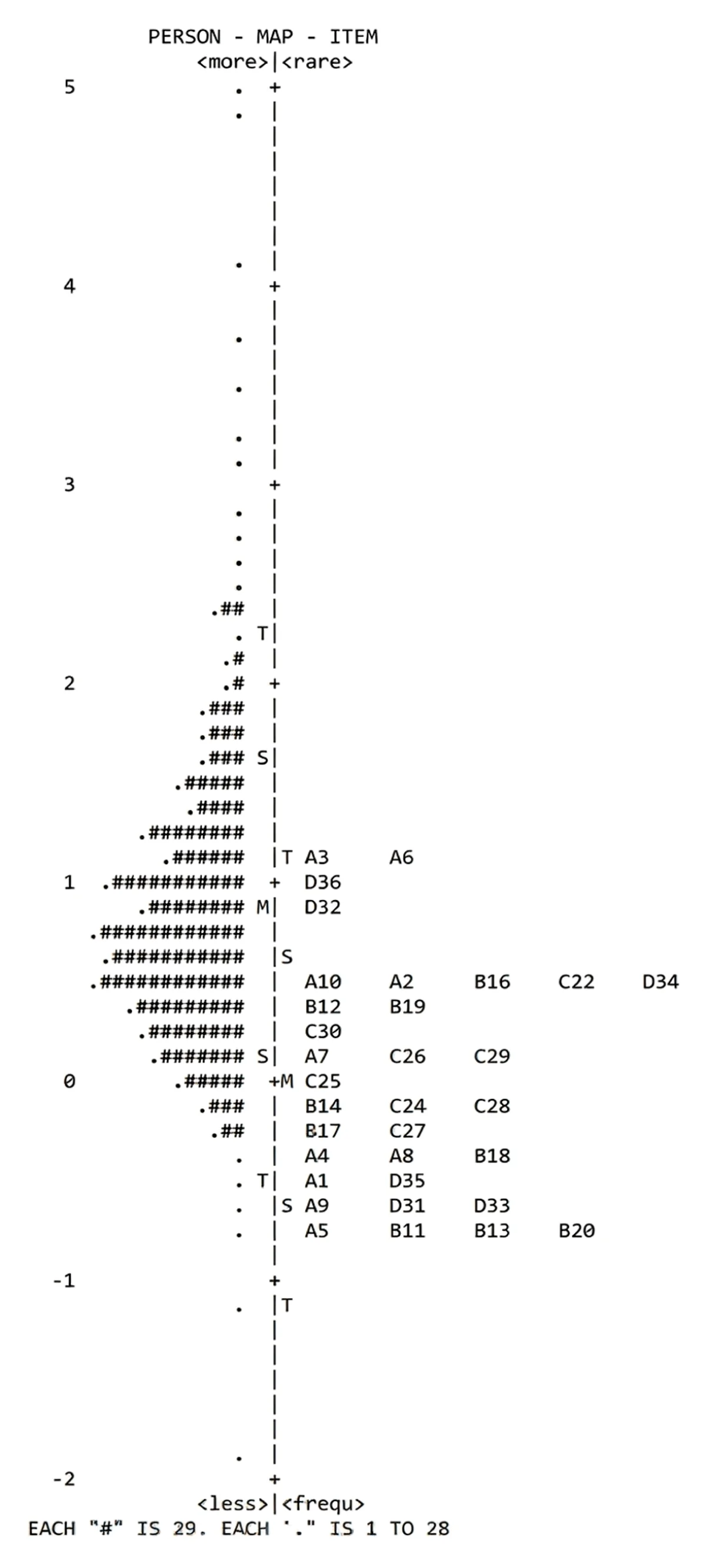
Person-Item Distribution Map (Wright Map) for the 33-Item Scale.

### 2.7 Data anonymisation and ethical issues

In the written consent form, respondents were given an explanation of the purpose of our research, that participation was voluntary, and that confidentiality was guaranteed in accordance with the Central Statistics Agency’s regulations on the implementation of sectoral statistics. Research involving human participants was reviewed, and we obtained ethical approval from the Research Ethics Committee of the Central Statistics Agency of the Province of Bangka Belitung (Number. B-626/BPS/1900/563/09/2024). Participants provided their written consent to participate in this study.

### 2.8 Existing use of data

Some measures from this dataset were used in a published study that can be accessed online here.

Pratama, D., Walidin, W., & Mahmud, S. (2023). Analisis Differential Item Functioning (DIF) Pada Skala Sikap Moderasi Beragama Siswa. Proyeksi. https://doi.org/10.30659/jp.18.1.116-131

## (3) Dataset description and access

### 3.1 Repository location

The dataset described in this study is publicly available. The dataset can be accessed via the Figshare repository. https://doi.org/10.6084/m9.figshare.30529121.

### 3.2 Object/file name

A Dataset of Religious Moderation Dataset of Senior High School Students.

Google Form Codebook. A Dataset of Religious Moderation Dataset of Senior High School Students.

### 3.3 Data type

Processed data, online survey items.

### 3.4 Format names and versions

.csv, .pdf

### 3.5 Language

The dataset and related files are stored in English. The survey items in their original language, along with their English translations, are provided in Appendix 1.

### 3.6 License

The dataset is released under the Creative Commons Attribution 4.0 International License (CC BY 4.0). This license allows users to copy, distribute, transmit, and adapt the material for any purpose, including commercial use, provided that appropriate credit is given to the original authors and the source of the dataset. Users may modify or build upon the dataset without restrictions on the license applied to derivative works, as long as proper attribution to the original dataset is maintained.

### 3.7 Limits to sharing

The dataset described in this study is publicly available.

### 3.8 Publication date

The date the dataset was published in the repository figshare was created on 04/11/2025.

## (4) Reuse potential

The dataset presented in this study offers significant potential for reuse across various academic and practical domains. Primarily, it serves as a high-quality resource for researchers in educational psychology and psychometrics who wish to conduct further advanced statistical analyses. Given that the data has undergone rigorous curation and validation through Rasch modeling, it provides a solid foundation for cross-validation studies or for comparing attitudes toward religious moderation across different geographical regions and demographic groups in Indonesia. Researchers may utilize both raw and processed data to examine complex relationships between students’ socio-demographic backgrounds and their religious moderation profiles.

Beyond psychometric evaluation, this dataset holds substantial value for the sociology of religion and educational policy development. The comprehensive nature of the four dimensions measured (national commitment, tolerance, anti-violence, and accommodation of local culture) allows for multifaceted exploration of religious extremism risks among the youth. Policymakers and educational practitioners can leverage this dataset to design evidence-based preventive programs and curricular interventions aimed at fostering a more inclusive and moderate religious outlook in schools. Furthermore, the high response rate and large sample size ensure that the data are robust enough to be integrated into larger meta-analyses or used as a baseline for longitudinal studies tracking changes in students’ attitudes over time.

## Appendix 1

### Scale Items

**Table:**

ITEM CODE	ORIGINAL LANGUAGE (INDONESIAN)	TRANSLATED VERSION (ENGLISH)	DIRECTION

A1	Pancasila sudah final sebagai ideologi Negara dan tepat diterapkan di Indonesia	Pancasila is final as the state ideology and is appropriate for Indonesia.	Favorable

A2	Berbagai permasalahan yang terjadi di Indonesia saat ini dikarenakan Pancasila kurang tepat sebagai ideologi negara	The various problems occurring in Indonesia today are because Pancasila is not a suitable ideology.	Unfavorable

A3	Di tengah persoalan yang melanda bangsa ini, keyakinan yang saya yakini lebih tepat dijadikan ideologi negara	Amidst the nation’s problems, the belief I hold is more appropriate to be the state ideology.	Unfavorable

A4	Agama yang saya yakini selaras dengan ideologi Pancasila	The religion I believe in is in harmony with the Pancasila ideology.	Favorable

A5	Nilai-nilai Pancasila perlu diamalkan dalam kehidupan sehari-hari untuk membangun komitmen kebangsaan	The values of Pancasila need to be practiced in daily life to build national commitment.	Favorable

A6	Islam merupakan agama mayoritas di Indonesia, sebaiknya Negara Islam ditetapkan sebagai ideologi Negara	As Islam is the majority religion in Indonesia, an Islamic State should be established as the state ideology.	Unfavorable

A7	Saya kurang bangga menjadi warga negara Indonesia karena banyak tertinggal dalam berbagai hal	I am not very proud to be an Indonesian citizen because we lag behind in many aspects.	Unfavorable

A8	Bagaimanapun kondisi bangsa, saya selalu berupaya memberikan kontribusi sesuai dengan kemampuan	Regardless of the nation’s condition, I always strive to contribute according to my abilities.	Favorable

A9	Saya lebih mengutamakan musyawarah dalam penyelesaian persoalan	I prioritize deliberation (musyawarah) in solving problems.	Favorable

A10	Bagi saya lebih memperjuangkan kepentingan pribadi dan keluarga daripada kepentingan kelompok bahkan Negara	For me, it is more important to fight for personal and family interests than for the interests of the group or the state.	Unfavorable

B11	Saya menerima adanya perbedaan suku, ras, agama, dan antar golongan	I accept the existence of differences in ethnicity, race, religion, and between groups.	Favorable

B12	Saya tidak akan mendukung pimpinan daerah/negara yang bukan berasal dari agama yang saya anut	I will not support a regional/national leader who is not from my religion.	Unfavorable

B13	Setiap warga negara memiliki hak yang sama dalam menjalankan agama yang diyakininya	Every citizen has the same right to practice the religion they believe in.	Favorable

B14	Dalam pergaulan saya hanya memilih teman yang seiman saja	In social interactions, I only choose friends who share my faith.	Unfavorable

B15	Saya meyakini ajaran agama saya paling benar, namun tetap menghargai ajaran agama yang lain	I believe my religion’s teachings are the most correct, but I still respect other religions.	Favorable

B16	Saya mendukung jika ada keluarga atau teman yang menutup diri dari orang yang berbeda agama/keyakinan	The existence of various religions in Indonesia has the potential to cause national division.	Unfavorable

B17	Saya tidak mempersoalkan pembangunan tempat ibadah agama lain yang sesuai dengan peraturan yang berlaku	I am willing to cooperate with anyone, regardless of their religious background.	Favorable

B18	Apapun bentuknya, cara-cara kekerasan tidak dibenarkan dalam kehidupan beragama	Religious differences are not a barrier to helping one another for the common good.	Favorable

B19	Saya berkeyakinan bahwa penganut agama lain punya tujuan untuk merubah keyakinan beragama saya	I feel uncomfortable when people of other faiths build their places of worship in my neighborhood.	Unfavorable

B20	Saya meyakini semua agama menganjurkan untuk senantiasa saling menghormati	I will not prohibit adherents of other religions from performing their worship.	Favorable

C21	Menurut saya, dalam menegakkan kebenaran perlu menggunakan kekerasan untuk memberikan efek jera kepada pelaku	I agree with coercive actions against groups considered heretical.	Unfavorable

C22	Penganut agama minoritas sebaiknya mengikuti keinginan dari penganut agama mayoritas	I am willing to engage in conflict with those who have different beliefs.	Unfavorable

C23	Saya tidak sepakat jika ada yang memberikan label sesat terhadap suatu agama atau aliran keagamaan	I choose dialogue to resolve differences in religious understanding.	Favorable

C24	Walaupun pernah melakukan penistaan agama, penganut agama mayoritas sebaiknya tidak bersikap sewenang-wenang kepada penganut agama minoritas	Violence in the name of religion cannot be justified under any circumstances.	Favorable

C25	Saya tidak mau memberikan donasi jika tidak memiliki keyakinan yang sama	When I see injustice, I think it is better to take matters into my own hands rather than leave it to law enforcement.	Unfavorable

C26	Menurut saya bahwa tindakan pengrusakan rumah ibadah dapat dibenarkan	In my opinion, the act of damaging places of worship can be justified.	Unfavorable

C27	Saya lebih mengutamakan musyawarah dalam penyelesaian persoalan keagamaan	I prefer to avoid any form of violence that could harm others.	Favorable

C28	Mengenai kasus penistaan agama, saya lebih memilih menyerahkan penanganannya kepada penegak hukum	I believe that every problem can be solved without violence.	Favorable

C29	Membubarkan kegiatan keagamaan lain boleh dilakukan apabila tidak sesuai dengan agama yang saya anut	Disbanding the religious activities of others is permissible if they are not in accordance with my religion.	Unfavorable

C30	Saya berusaha mengajak teman untuk melakukan tindakan kekerasan terhadap sekelompok orang yang melecehkan keyakinan saya	I try to persuade friends to commit acts of violence against a group of people who insult my beliefs.	Unfavorable

D31	Ajaran agama yang saya yakini memberikan ruang untuk menerima budaya atau tradisi baik yang sudah berlangsung lama di masyarakat	The teachings of my religion provide space to accept good cultures or traditions that have long existed in society.	Favorable

D32	Semua kebudayaan atau tradisi lokal yang berkembang di masyarakat bertentangan dengan ajaran agama yang saya yakini	All local cultures or traditions that develop in society are contrary to the teachings of my religion.	Unfavorable

D33	Saya selalu berusaha untuk menghargai dan menghormati setiap kebudayaan atau tradisi yang berkembang di Indonesia	I always try to appreciate and respect every culture or tradition that develops in Indonesia.	Favorable

D34	Menurut saya semua tradisi lokal yang berkembang di masyarakat tidak sesuai dengan ajaran agama yang saya anut	In my opinion, all local traditions that develop in society are not in accordance with the teachings of my religion.	Unfavorable

D35	Saya meyakini bahwa budaya atau tradisi lokal yang berkembang di masyarakat menjadi salah satu penguat persatuan bangsa	I believe that the local cultures or traditions that develop in society are one of the forces that strengthen national unity.	Favorable

D36	Musibah atau bencana yang terjadi pada bangsa ini adalah teguran dari Tuhan Yang Maha Esa karena mencampuradukkan ajaran agama dengan budaya atau tradisi lokal	Disasters or calamities that occur in this nation are a reprimand from God because people mix religious teachings with local culture or traditions.	Unfavorable
