# Supplementary material for: Religious Moderation Dataset of Senior High School Students in Indonesia
Source: J Open Psychol Data. 2026 Oct 12;14:5. doi: 10.5334/jopd.163 (PMC13674529; doi:10.5334/jopd.163)
Supplement: Supplementary file 1 [file jopd-14-163-pr1.pdf]

First Revision

Response to Reviewer

Response to Editor

Theoretical Framework: We have expanded the description of the four dimensions of religious moderation, providing clearer definitions and theoretical underpinnings for international readers. **Methodological Clarification**: We have added specific details regarding the stratified random sampling procedure, the random lottery system for participant incentives, and the technical measures taken (Google Forms' "Limit to 1 response") to prevent duplicate participation. **Technical Consistency**: We have resolved the discrepancies regarding item C27 and updated the Codebook to include all demographic variables (parental employment, school status, etc.) and computed total scores to match the raw dataset.

Statistical Reporting: We have provided justifications for the use of Outfit MNSQ and improved the interpretation of the Wright Map as requested. **Administrative Updates**: The licensing terms have been clarified to CC BY 4.0, and the "Reuse Potential" section has been strengthened with more specific use cases. **Item Development**: We have added information regarding the item generation process, expert validation, and the back-translation procedure used for the English version.

Reviewer 1

Point 1 (Theoretical underpinnings): Thank you for this suggestion. We have expanded the theoretical explanation of the four dimensions of religious moderation (national commitment, tolerance, anti-violence, and accommodation of local culture). The revised manuscript now includes a detailed conceptual explanation of each dimension and its relevance in the Indonesian context. I have also read and understood the research by Basedau et al., (2021) as relevant to include in this section.
Location in manuscript: Section 1.1.

Point 2 (Item C27 and Demographics): We apologise for this oversight. We have corrected the wording of item C27 in both the manuscript and the code book to ensure consistency. The final version used in data collection was the statement, "**I prefer to avoid any form of violence that could harm others**." In addition, we have updated the code book and manuscript to include clear value labels for all demographic variables (e.g., parents' occupation, school status).

Point 3 (Methodological concerns - Sample size): We understand and acknowledge concerns regarding simplicity. A large sample size was deliberately chosen to maximise statistical power in the Rasch model and ensure a representative distribution across the diverse types of schools in Bangka Belitung Province. We have added justification for this in the "Sampling" section. Regarding criterion validity, although it is not the main focus of this data paper, we have highlighted this as a priority for future studies in the "Potential for Reuse" section.
Location in manuscript: Section 2.4.

Point 4 (Reuse potential): In accordance with the reviewer's recommendations, we have simplified the "Potential for Reuse" section. We removed the less specific points (1, 2, and 5) and expanded points 3 and 4 to explain specific use cases involving exploratory demographic analysis and cross-context scaling adjustments.

Reviewer 2

Point 1 & 9 (Duplicates & Software): We have clarified in the Methods section that Google Forms was used with the "Limit to 1 response" setting to prevent duplicates. The mention of Qualtrics in the codebook was a clerical error and has been corrected to Google Forms.

Point 2 & 3 (Strata & Souvenirs): We have added details regarding the stratification factors (geography and school type) and clarified that the 100 souvenir recipients were chosen via a random lottery. **Location in manuscript**: Section 2.4.

Point 4 (The presence of the words "In Additions..." in the sampling and data collection section):
It has been corrected. That section now focuses more on explaining that the instrument is indeed intended to measure perceptions of religious moderation using a 5-point Likert scale.

Point 5 & 6 (Statistics & Wright Map): We have added a justification for our focus on Outfit MNSQ and provided a more in-depth interpretation of the Wright Map, acknowledging the instrument's high precision for average respondents.
Location in manuscript: Section 2.6.

Point 7 & 8 (Dataset Consistency): The Codebook has been updated to include all demographic variable labels found in the raw data (Parents' employment, School type, etc.). We also clarified that the dataset intentionally contains raw item-level responses only, without computed variables such as MB_Total, A_Total, B_Total, C_Total, or D_Total. These values can be calculated by users if needed for their analyses. The codebook has been updated accordingly to avoid confusion.

Point 9 (Contradictions between Google Forms and Qualtrics)
We apologise for this inconsistency and thank the reviewer for their thoroughness. We have revised the manuscript and appendix materials to ensure absolute consistency regarding the data collection platform. Data collection is conducted exclusively using Google Forms. The mention of 'Qualtrics' in the previous Codebook was a technical/writing error, and the Codebook has now been corrected to reflect the fact that the survey is conducted via Google Forms.

Point 10 (License): Thank you for identifying this inconsistency. The dataset in the repository is licensed under the Creative Commons Attribution 4.0 International License (CC BY 4.0). In the previous version of the manuscript, the description incorrectly suggested a ShareAlike requirement. We have revised Section 3.6 (License) to accurately reflect the CC BY 4.0 license and clarified that users are allowed to modify and reuse the dataset, provided that proper attribution to the original authors is given.
Location in manuscript: Section 3.6.

Point 11 (Item Generation): A new paragraph has been added to describe the item generation process, expert validation, and the back-translation method used for the English version.

Location in manuscript: Section 2.5.

Second Revision

Response to Editor

Editor's Comment 1:

Authors responded that "In addition, we have updated the codebook and manuscript to include clear value labels for all demographic variables (e.g., parents' occupation, school status). → I am unable to identify these changes in the provided materials..."

Author's Response:

We sincerely apologize for any confusion. We would like to clarify that the updates to the demographic variables (specifically requested by the reviewer regarding parents' employment status, type of school, and school status) have been fully implemented in **Part 1: Identity and Demographic Variables of the revised Codebook.** This section has been revised based on the corrections provided by Reviewer 2.

Below is a comparison table to highlight the specific additions made to the codebook to align with the "Raw Data.xlsx" file:

Qualtrics Codebook: Religious Moderation Survey

Part 1: Identity and Demographic Variables

Variable Name	Variable Label (Description)	Measurement Level	Value Labels	Missing Code
Timestamp	Time of survey completion	Scale (Time)	(Date and time data)	(Blank)
Nama	Respondent's Name (Optional)	Nominal	(Free text)	(Blank)
Gender	Respondent's Gender	Nominal	1 = Male 2 = Female	(Blank)
Agama	Respondent's Religion	Nominal	1 = Islam 2 = Christian 3 = Catholic 4 = Hindu 5 = Buddhist	(Blank)

Previous Codebook

Google Form Codebook: Religious Moderation Survey

Part 1: Identity and Demographic Variables

Variable Name	Measurement Level	Value Labels	Missing Code
Respondent's Gender	Nominal	1 = Male 2 = Female	(Blank)
Respondent's Religion	Nominal	1 = Islam 2 = Christian 3 = Catholic 4 = Hindu 5 = Buddhist	(Blank)
Parents' employment status	Nominal	1 = Working 2 = Not Working	(Blank)
Type of School	Nominal	1 = High School 2 = Islamic High School 3 = Vocational High School	(Blank)
School Status	Nominal	1 = Public School 2 = Privat School	(Blank)
City of Origin	Nominal	Bangka<br>West Bangka<br>South Bangka<br>Central Bangka<br>Belitung<br>East Belitung<br>Pangkalpinang	

Revised Codebook

Editor's Comment 2:

"Please also include the original language items in the appendix, presented in a table alongside their translated versions for transparency."

Author's Response:

As requested, we have added a new Appendix containing a comprehensive table that presents the original Indonesian items alongside their English translations

Editor's Comment 3:

"The sample description should be expanded. Please include mean age and age range, gender distribution, religious affiliation distribution".

Author's Response:

We have expanded the sample description in the revised manuscript (see Section 2.4). We have also added a demographic table (Table 1) to the manuscript to make this information more accessible to readers.

Editor's Comment 4:

The "Reuse Potential" section should be revised for clarity and readability. Please replace the current bullet-point format with well-structured paragraphs and ensure a coherent narrative flow.

Author's Response:

As suggested by the editor, we have revised the Reuse Potential section. The previous bullet-point format has been replaced with well-structured paragraphs to ensure a better narrative flow and readability.

Editor's Comment 5:

Abstract: The abstract should be revised to provide a more precise and informative description of the sample, including mean age, age range, and gender distribution. In addition, the time of data collection should be specified. The abstract should place greater emphasis on the dataset itself rather than on methodological details; for example, the Rasch analysis should be mentioned only briefly without detailed references to specific items, and technical terminology should be kept to a minimum. Finally, the reuse potential should be more clearly articulated by focusing less on methodological quality assurance and more on the relevance of the dataset, explicitly outlining why the data are important, who can use them, and for which types of research or applications.

Author's Response:

Thank you for your constructive feedback and the opportunity to revise our manuscript. We have carefully revised the abstract and the section on potential applications to reflect your suggestions. We have revised the abstract as shown below and have incorporated it into the manuscript.

Third Revision

Response to Editor

Comment 1: Abstract clarity

"This sentence is not quite clear…"

Response:

Thank you for this valuable suggestion. We have revised the sentence in the abstract for clarity and conciseness. We have also revised the percentage values. The sentence now reads:

"*This paper presents a large-scale dataset on adolescent attitudes toward religious moderation, validated using the Rasch model to ensure high psychometric quality and reliability*."

Previous	Revised	
**Abstract**<br>This article presents a comprehensive dataset on attitudes towards religious moderation among 4.197 secondary school students in the Bangka Belitung Islands Province, Indonesia. Data collection was conducted online via Google Forms between November and December 2022. The participants represent a homogeneous developmental group in late adolescence, with an age range generally between 15 and 19 years and a mean age of approximately 16.8 years. The gender distribution of the sample comprised 1.627 males ~~(38.8%)~~ and 2.570 females ~~(61.2%)~~. This dataset measures four key dimensions of religious moderation (national commitment, tolerance, anti-violence, and accommodation of local culture). Although the data have been validated using the Rasch model to ensure high psychometric quality and reliability, the focus of this contribution is the availability of robust, large-scale empirical evidence regarding adolescent attitudes. This dataset is a valuable resource for researchers in the fields of educational psychology, the sociology of religion, and policymakers. It can be utilized to map the risks of extremism, design evidence-based character education curricula, or serve as a baseline for longitudinal studies on religious harmony among the younger generation in multicultural societies.<br>**Maehler, Débora**<br>"This sentence is not quite clear yet; I suggest the following: „This paper presents a large-scale dataset on adolescent attitudes, validated using the Rasch model to ensure high psychometric quality and reliability.„	**Abstract**<br>This article presents a comprehensive dataset on attitudes towards religious moderation among 4.197 secondary school students in the Bangka Belitung Islands Province, Indonesia. Data collection was conducted online via Google Forms between November and December 2022. The participants represent a homogeneous developmental group in late adolescence, with an age range generally between 15 and 19 years and a mean age of approximately 16.8 years. The gender distribution of the sample comprised 1.627 males and 2.570 females. This dataset measures four key dimensions of religious moderation (national commitment, tolerance, anti-violence, and accommodation of local culture). Although the data have been validated using the Rasch model to ensure high psychometric quality and reliability, the focus of this contribution is the availability of robust, large-scale empirical evidence regarding adolescent attitudes. This dataset represents a valuable resource for researchers in the fields of educational psychology, the sociology of religion, and policymakers. It can be used to identify potential risks of extremism, design evidence-based character education curricula, or serve as a baseline for longitudinal studies on religious harmony among the younger generation in multicultural societies.	

Comment 2: Add reference to Table 1

Response:

We have added a reference to Table 1 in the manuscript. The sentence now reads:

"*The demographic characteristics of the participants are presented in Table 1*."

Comment 3: Remove redundant paragraph

Response:

We agree with the reviewer. The paragraph describing the demographic characteristics in detail has been removed to avoid redundancy, as this information is already clearly presented in Table 1.

Previous	Revised	

Religion	Islam	3.512	83.7%
	Buddha	316	7.5%
	Protestant	157	3.7%
	Catholic	128	3.0%
	Confucianism	82	2.0%
	Hindu	2	0.0%
Parents' Employment Status	Working	2.627	62.6%
	Not Working	1.57	37.4%
Type of School	High School	1.921	45.8%
	Islamic High School	1.551	37.0%
	Vocational High School	725	17.3%
School Status	Public	2.994	71.3%
	Privat	1.203	28.7%
City of Origin	Bangka	780	18.6%
	West Bangka	825	19.7%
	South Bangka	572	13.6%
	Central Bangka	368	8.8%
	Belitung	660	15.7%
	East Belitung	431	10.3%
	Pangkalpinang	561	13.4%

In the Indonesian educational context, this demographic represents a homogeneous developmental stage known as late adolescence, with a typical age range of 15 to 19 years. The gender distribution shows a higher representation of female students, totaling 2.570 (61.2%), while male students accounted for 1.627 (38.8%) of the sample. Regarding religious affiliation, the dataset reflects the diverse religious landscape of the Bangka Belitung Islands Province. The majority of the respondents identified as Muslim (83.7%), followed by Buddhists (7.5%), Protestants (3.7%), Catholics (3.1%), and Confucians (2.0%). A small minority identified as Hindu (0.05%).

Maehler, Débora
This paragraph is not necessary, as the sample is already well described in Table 1; otherwise, it would be redundant.

Maehler, Débora
Suggest to remove this paragraph

	Confucianism	82	2.0%
	Hindu	2	0.0%
Parents' Employment Status	Working	2.627	62.6%
	Not Working	1.57	37.4%
Type of School	High School	1.921	45.8%
	Islamic High School	1.551	37.0%
	Vocational High School	725	17.3%
School Status	Public	2.994	71.3%
	Private	1.203	28.7%
City of Origin	Bangka	780	18.6%
	West Bangka	825	19.7%
	South Bangka	572	13.6%
	Central Bangka	368	8.8%
	Belitung	660	15.7%
	East Belitung	431	10.3%
	Pangkalpinang	561	13.4%

The sample size for this study was not determined based on power analysis (such as 1-β), which is commonly used to test inferential hypotheses. Instead, because the main objective of this study was to describe the level of religious moderation among students, the sample size was determined based on the desired estimation precision. This survey used the Cocran (Tengeh & Talom, 2020) sample estimation formula for large populations, with a confidence level of 95% (equivalent to Z = 1.96) and a margin of error (e) of 5% (0.05), assuming the most conservative population variance (p = 0.5). The minimum sample size required is 385. Based on these calculations, the minimum sample size required to achieve ± 5% precision is 385 participants. Our final sample size of 4197 participants exceeded this minimum threshold, ensuring that our findings are highly precise and can be generalized with 95% confidence.

Comment 4: Add reference to Table 2
Response:
We have added a reference to Table 2 in the manuscript. The sentence now reads:
"*The measurement instrument comprised 36 items across four dimensions of religious moderation. The full list of item statements, along with their dimensional classification and directional coding, is provided in Table 2*."

Comment 5: Add reference to Table 3
Response:
We have added a reference to Table 3 in the manuscript. The sentence now reads:
"*The summary of the psychometric analysis results is presented in Table 3*."

Comment 5: Add reference to Table 4
Response:
We have added a reference to Table 4 in the manuscript. The sentence now reads:
"*A comparison between the initial and final psychometric analysis results is presented in Table 4*."

Comment 6: Add note about translation availability
Response:
We have added a clarification in Section 3.5 (Language) stating that:
"*The English translations of the survey items are also provided in Appendix 1*."

[Attention: This correction was suggested by the editor because the sentence may be misleading, given that the original survey language was not English: "The survey items in their original language, along with their English translations, are provided in Appendix 1."]

In addition to addressing all revisions and comments highlighted in the manuscript, the authors have also conducted a comprehensive proofreading of the entire document. This process was carried out to ensure clarity, consistency, and accuracy in language, as well as to improve the overall readability and quality of the manuscript.
